# Translational Acceleration, Rotational Speed, and Joint Angle of Patients Related to Correct/Incorrect Methods of Transfer Skills by Nurses

**DOI:** 10.3390/s18092975

**Published:** 2018-09-06

**Authors:** Chingszu Lin, Masako Kanai-Pak, Jukai Maeda, Yasuko Kitajima, Mitsuhiro Nakamura, Noriaki Kuwahara, Taiki Ogata, Jun Ota

**Affiliations:** 1 Research into Artifacts, Center for Engineering (RACE), The University of Tokyo, Chiba 277-8568, Japan; ogata@race.u-tokyo.ac.jp (T.O.); Ota@race.u-tokyo.ac.jp (J.O.); 2 Faculty of Nursing, Kanto Gakuin University, Yokohama 236-8501, Japan; kanaipak@kanto-gakuin.ac.jp; 3 Faculty of Nursing, Tokyo Ariake University of Medical and Health Sciences, Tokyo 135-0063, Japan; jukai@tau.ac.jp (J.M.); kitajima@tau.ac.jp (Y.K.); m-nakamura@tau.ac.jp (M.N.); 4 Department of Advanced Fibro-Science, Kyoto Institute of Technology, Kyoto 606-8585, Japan; nkuwahar@kit.ac.jp

**Keywords:** human movement, inertial sensors, motion capture, patient transfer skill

## Abstract

Currently, due to shortages in the nursing faculty and low access to actual patients, it is difficult for students to receive feedback from teachers and practice with actual patients to obtain clinic experience. Thus, both evaluation systems and simulated patients have become urgent requirements. Accordingly, this study proposes a method to evaluate the nurse’s transfer skill through observation from the patient. After verifying the proposed method, it will be integrated with a robotic patient as a future work. To verify if such an evaluation is practical, a checklist comprising 16 steps with correct and incorrect methods was proposed by the nursing teachers. Further, the evaluation parameters were determined as translational acceleration, rotational speed, and joint angle of patient. Inertial sensors and motion capture were employed to measure the translational acceleration, rotational speed, and joint angle. An experiment was conducted with two nursing teachers, who were asked to carry out both correct and incorrect methods. According to the results, three parameters reveal the difference for a patient under correct/incorrect methods and can further be used to evaluate the nurse’s skill once the thresholds are determined. In addition, the applicability of inertial sensors is confirmed for the use of robot development.

## 1. Introduction

Nowadays, with the increasing need for nursing care at hospitals and care centers [1], school education plays an important role in imparting patient handling skills. However, little access to actual patients and a shortage of nursing faculty members are demerits at schools, affecting the learning quality of the students. According to a report by the American Association of Colleges of Nursing [2,3,4], the problem of faculty shortage is rising and contributes to the teacher–student ratio in clinical courses becoming smaller. Further, studies [5,6] have claimed that larger class sizes lead to insufficient supervision by teachers and less feedback. In such situations, the students are unable to learn from mistakes and further restructure their skills [7,8]. The other problem is that the nursing students have little access to practice with actual patients [9], due to safety and ethics concerns. Accordingly, after starting a career at a hospital, they need to take a few years to accumulate clinic experience to be considered experts [10,11]. Thus, proposing a robot to simulate actual patients and also an evaluation system of students’ performance have become urgent requirements. 

**Figure 1:**
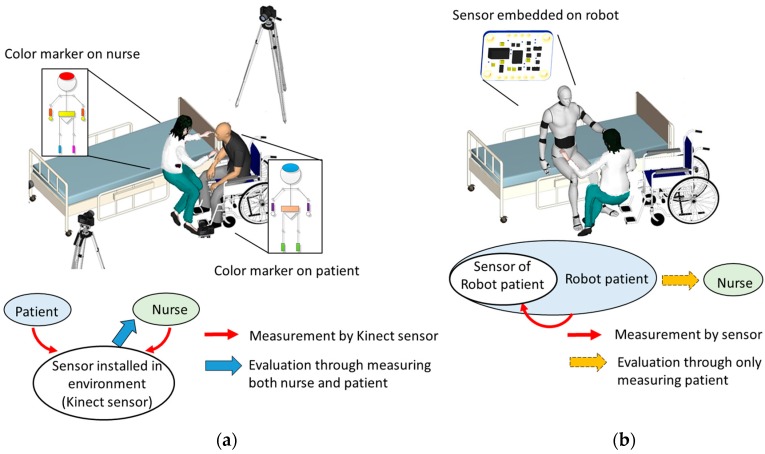
Evaluation system of transfer skill (**a**) our previous work (**b**) final goal.

According to this background, our previous work proposed an evaluation system using Kinect sensors for nursing students to learn transfer skills [12]. Further, a robot was developed to simulate various patients during transfer [13]. However, due to the proposed system observing both the nurse and patient, complex sensor settings on learners (i.e., color markers) and the environment (i.e., Kinect sensor) are inevitable, as shown in Figure 1a. Therefore, our final goal is to integrate the evaluation system within the robot. In this way, any sensor setting and camera on learners and environment can be avoided, as shown in Figure 1b. One of the solutions is to propose an evaluation system that can evaluate the nurse’s skill only by measuring the patient without nurse. The final goal can be achieved by installing the sensors on a robot patient to observe itself. However, the feasibility of such evaluation method is still uncertain because the influence on the patient caused by the nurse’s skill is unknown. Therefore, the motivation of this study is to realize the interaction between the nurse’s skill and the patient as an initial stage.

With the advancement of technology, many evaluation systems employing different sensors have been proposed. For example, a learning interface for dancing, with immediate feedback by employing motion capture technology, was proposed in [14]. Other research has implemented a system using an underwater camera, wearable LED marker, and pressure sensor to analyze the skill of a swimmer [15]. In addition, an evaluation method using inertial sensors for golf training was proposed [16,17]. Furthermore, the arm movement of novice and expert baseball players was observed in [18]. In addition, a study investigated the difference between expert and novice piano players with respect to upper-limb movement through both EMG and a camera [19]. Despite these studies proposing different methods to evaluate the learners’ performance, the effects of the sensor on the learner and environment are inevitable. For instance, the color marker [12] and motion capture marker [14] need to be attached to the trainee’s body, and the inertial sensors must be set on the player’s golf club [16,17]. Further, the motion capture camera and Kinect sensor employed in our early work required time consuming calibration and installation [12,13,14]. The aforementioned reasons make the proposed systems have low applicability in cases requiring portability or having a large number of students. 

Transfer skill is a target to improve the learning of nursing students, due to its importance in a patient’s daily life and the difficult body mechanics between the nurse and patient. However, to use an evaluation system that only employs the measurement of a patient is challenging because the nurses who actively conduct the task are not observed. Therefore, the mutual interactions between the nurse and patient during the transfer task becomes crucial to determine whether such an evaluation is feasible. Some previous studies investigated the mutual interaction between individuals during the different tasks. For example, in [20], the coupling between a speaker’s and a listener’s eye movements was observed to realize the mutual interaction during a speech. In addition, a collaborative object-lifting task with physical interaction between a human and robot was observed, including the force and trajectory [21]. Furthermore, a real-time dancing game facilitated recognition of the dancer’s moves and gives a simultaneous response through a virtual partner who performs simultaneously with the dancer on a monitor [22]. Other research investigated how the robot adapting its motion depends on the human’s movement while being assisted to stand up [23]. Furthermore, in [24,25], the researchers discovered the relationship between a patient’s satisfaction and nurse’s caring, revealing that communication skills and medical-technology have a strong relationship with patient satisfaction. However, although the researchers in [24,25] found the relationship between mental satisfaction of patient and nurse’s care, the physical interaction of the patient has not be considered. The patient handling skills require mutual body movements between the nurse and patient, and the physical interaction of the patient is the most directive and instant impact caused by the nurse. Furthermore, for cooperative tasks between two individuals in [22,23], only the correct cooperative manner was observed, while the incorrect interaction in the incorrect manner has not been considered. However, the influence on the patient under inappropriate interaction of nurses is essential because this influence is strongly related to the safety of the patient [26].

Further, little access to actual patients makes it difficult for students to obtain clinic experience. To overcome this problem, a simulated robot patient was developed to simulate a real patient, allowing the students to practice [27]. For example, Gerling et al. [28] developed a simulator for prostate conditions that can reproduce abnormal conditions for diagnosis training. Further, limb robots enabling the simulation of symptoms caused by spasticity and rigidity were developed for training physical therapists [29,30]. In addition, a simulator was developed for a student to learn how to manage airway difficulties. Furthermore, full-body robots were developed to imitate chaotic emotions for injection training [31], and also clothe-changing training [32]. Moreover, Takanobu et al. constructed a robot with an oral structure to reproduce oral cavities and bleeding for clinical training [33]. However, even though the developed robots can simulate patients, these studies have not considered an evaluation system. Therefore, teachers are still required for evaluating the students’ performance. 

To propose the evaluation system for transfer skill using only patient measurement, we determined the movement of the patient to evaluate a nurse’s skill, because the transfer skill involves many physical interactions between the nurse and patient (e.g., mutual hugging and turning). Accordingly, an assumption is raised, considering that the correct/incorrect methods used by nurses will cause different influences on the patient’s movement. The first purpose of this study is to prove this assumption by investigating the interaction between the nurse’s correct/incorrect methods and the patient’s movements. If the different influence on a patient under the correct/incorrect methods of the nurse can be obtained in the present study, the evaluation system can be achieved while the threshold is settled in a further step. In order to determine the parameters for measuring, this study proposed a checklist written with both correct and incorrect methods, based on discussions with nursing teachers. Furthermore, through an observation of patients and the content of the checklist, the patient’s movements under correct/incorrect may affect the following three parameters of patients: translational acceleration, rotational speed, and joint angle, and those data are measured by inertial sensors and motion capture. An experiment was conducted with two nursing teachers. They were asked to carry out the transfer task through both correct and incorrect methods and were also asked to take turns simulating a patient. Finally, the patient was measured and analyzed to check whether a difference can be revealed by the translation acceleration, rotational speed, and joint angle when the nurse uses the correct and incorrect methods.

The second purpose of this study is the advance preparation of development, which verifies the applicability of the inertial sensors to be developed for the robot. During the experiment, the translational acceleration and the rotational speed were measured by the inertial sensor, which will also be developed in a robot in the further step. The joint angle was measured by motion capture, which will be replaced by encoders directly attached to the robot joint. However, the inertial sensor has relatively high noise, low robustness and accuracy, and can also be affected by magnetic fields and gravity [34,35,36]. Therefore, if the measured data from inertial sensors are applicable to evaluate a nurse’s skill, the inertial sensor can be further used in the robot development.

The remainder of this paper is organized as follows. In Section 2, the proposed method of both the patient transfer task and measuring method are introduced. The experiment, results, and discussion are presented in Section 3 and Section 4. Finally, conclusions and future work are described in Section 5.

## 2. Proposed Method

### 2.1. Patient Transfer skill and Checklist

**Figure 2:**
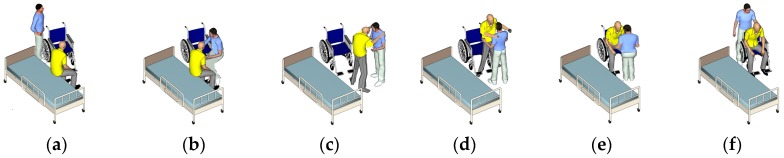
Patient transfer skills of (**a**) sitting on the bed; (**b**) mutual hugging; (**c**) standing up; (**d**) pivot turning; (**e**) sitting down on the wheelchair; and (**f**) final posture adjustment.

Patient transfer is one of the most difficult but indispensable tasks for nurses to take care of patients in hospitals or homes [31]. During daily life, the transfer skill is required to assist the patients in moving to the toilet or bathroom. Further, physical interaction and mutual cooperation between patients and nurses occur during the transfer, such as mutual hugging and turning together [32]. Furthermore, this skill requires many body mechanics (e.g., position, posture, and method of movement) to ensure patient comfort and prevent accidents. The main steps during patient transfer are presented in Figure 2. The steps are sitting on the bed, mutual hugging, standing up, pivot turning, sitting down on the wheelchair, and final posture adjustment. Owing to the transfer skill involving the entire body movement of a patient and nurse in each step, as shown in the Figure 2, the patient’s movement becomes key to evaluating the nurse’s skill. However, only the movement of a patient to evaluate the nurse’s skill still remains uncertain, because the relationship between the nurse’s skill and the movement of the patient has not be observed. Therefore, to realize such relationships, the influence of a patient’s movement while the nurse carries out this under correct and incorrect methods should be investigated. Based on our early work [16], this study proposed a new checklist with both the correct and incorrect ways through a discussion with nursing teachers, as shown in Table 1. Further, the steps that could cause the difference in influence on the patient between correct and incorrect methods were listed in the checklist.

The incorrect ways were determined as the common mistakes of nurses, which allow us to represent the conventional situations of inappropriate care at hospitals. Of course, other uncommon incorrect ways do exist, but this study does not take these into account at the present stage. Also, all the correct/incorrect methods are created referring to clinical experience and nursing materials. Due to the strong connective relationship between steps, when the incorrect ways were conducted, some influence of the patient occurs at not only the present step, but also at the other steps. For example, the placement of the wheelchair at No. 1 and No. 3 will have an influence on the patient during the pivot turning step No. 12. The checklist contains 16 evaluation items, and 12 items were written with incorrect methods. 

### 2.2. Patient’s Movements Related to Transfer Skill 

The movement of a patient is a key to evaluate the transfer skill, because the transfer skill involves the entire body movement and physical interaction between the nurse and patient. Therefore, what kind of movements occur during the transfer should first be investigated. The parameters that are able to reflect the patient’s movement under incorrect methods must then be determined.

According to the observation of the patient during the transfer and the discussion with experienced nursing teachers, the patient’s movements include the directional and rotational motions. Each motion also results from the changing of the joint movement. Accordingly, all movements of the patient during the transfer process can be revealed through translational/rotational motions and joint variation. Translational and rotational motions enable the representation of the comprehensive information of the patient’s movement. Joint variation is a fundamental element contributing to the movements; therefore, the movement in the local joint can be realized by investigating the variation of each joint. The definitions and explanations are as follows.

● Comprehensive Movement of the Patient

During the transfer skills, the patient’s movements involve translational and rotational motions. Those motions are able to exhibit the general physical properties.

- *Translational Motion*

Translational motion represents a dynamic movement in a linear direction in three-dimensional space. For example, in step No. 11, when the nurse assists the patient to stand up from the bed, the trunk of a patient moves in an upward direction. Also, in step No. 15, when assisting the patient to sit down on the wheelchair, the downward motion of the patient can be found.

- *Rotational Motion*

Rotational motion is different from translational motion, which moves in a linear path. During rotation, a point or a part of rigid body is fixed, while other parts of rigid body move relative to it. Rotational motion enables us to deal with a patient’s movement, such as pivot turning in step No. 12. 

● Local Movement of the Patient

Different from the translational and rotational motion, the joint variation cannot reveal the general information of how a rigid body moves in a space, but joint variation enables us to provide the local information of each partial body. With the local information of the joint, the details of posture changing can be analyzed.

- *Joint Variation*

Each variation of the joint represents the posture changing for a specific body part. For example, in step No. 7, when the nurse places the patient’s arms on his/her shoulder to conduct the mutual hug, the joint angle of patient’s shoulder changes.

### 2.3. Measured Parameters

The data capable of measuring the motions need to be determined after decomposing the patient’s movements during transfer. Various types of data can represent the different motion properties. However, in this study, we intend to use fewer parameters to decrease the system complexity and effectively evaluate the nurse’s skill. In this way, the development of the robot can become simpler because less sensors are required to be used.

● Translational Acceleration

Acceleration is determined as a crucial parameter to represent the translational motion. Acceleration in physics enables impulsive movement in a linear path, with the speed changing significantly with respect to time. Therefore, the index of acceleration enables us to exhibit the dynamic movement caused by the forces exerted by the nurses. For example, when the nurse assists the patient in standing up or sitting down, the translational acceleration of the patient can reveal how well the nurse has supported the patient (i.e., strongly, weakly).

● Rotational Speed

Rotational speed is determined from the data that represent the rotational motion. It reveals the dynamic condition of rotation. The numerical value represents different degrees of speed. For instance, the rotational speed along the vertical axis of the trunk represents the lateral turning of the trunk. Likewise, the rotation speed along the horizontal axis of the hip joints reveals the forward/backward movement of the upper body.

● Joint Angle

The human skeleton comprises different joints (e.g., hip, shoulder). Accordingly, the skeletal information of the posture can be realized by measuring the joint angle of the patient. For example, the flexion angle of the knee will decrease when a patient moves from sitting to standing posture. Although most postures of a patient can be represented through the joint angles, acceleration and rotational speed are necessary to exhibit the dynamic condition of the patient.

### 2.4. Sensors

#### 2.4.1. Determination of Sensor

The first requirement for determining the sensors is that the sensors must be able to measure the data introduced above. Secondly, because the purpose of the sensor is to measure the patient’s movement, the installation of the sensors should avoid affecting the movement of the patient. Furthermore, the sensor should be capable of being developed on the robot, or our final goal cannot be achieved. 

To satisfy the above requirements, inertial sensors and motion capture are employed in this study. The inertial sensors are used to measure the translational acceleration and rotational speed data. The inertial sensors will also be placed on the robot during its development in order to measure these movements. Motion capture is utilized to measure the joint angle of the patient, although the complicated setting of the environment is a disadvantage. Motion capture is used because the motion capture markers are attached on the patient to evaluate the joint angle, which have lesser influence on the patient’s movement than angular sensors that are required to be attached on the joints directly. In addition, this study first verifies whether the joint angle of the patient is useful in the evaluation of the skill of the nurse; if this is confirmed, the robot joint will be developed with the encoder in the future. 

The product TSND121 (ATR-Promotions Co., Ltd., Kyoto, Japan) was selected as the sensor due to its compact size of 37 mm (W) × 46 mm(H) × 12 mm(D) and light weight of 22 g, which can be easily installed on the body and barely affects physical movement. In addition, the joint angles were measured by motion capture cameras (Kestrel; Motion Analysis, Co., Santa Rosa, CA, USA) through the motion capture markers. Furthermore, software (Cortex; Motion Analysis, Co., Santa Rosa, CA, USA) was used to edit the recording videos with the labels and links on the joint. With the data of the joint as the input, SIMM (Cortex; Motion Analysis Co., Santa Rosa, CA, USA) enables the calculation of the musculoskeletal model such as the joint angle in any body position.

#### 2.4.2. Installation on Patient

The steps that will incorrectly influence the patient are presented in the checklist. However, the patient transfer skill involves the entire body movement of the patient. For example, the movement of standing up includes not only the motion of the feet but also the trunk. Therefore, different degrees of influence will occur on different body parts during these steps. It is important to determine where the most significant difference occurs. Accordingly, the entire body of the patient was measured, and then the location of the most significant influence on the patient was determined based on the obtained data. 

**Figure 3:**
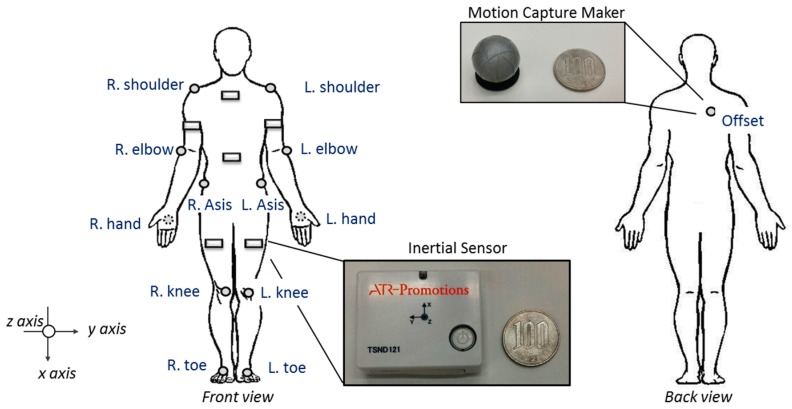
Installation of inertial sensors and motion capture markers on the patient.

The human body consists of four limbs and a trunk; six inertial sensors were installed on both upper/lower limbs and the trunk to measure the entire body. The inertial sensors were installed on both arms and on both thighs. With regard to the trunk, the waist and chest are located at high and low positions because they exhibit different influences of the movement. The waist is the center of the human body and it represents the entire body movement; therefore, it can represent the patient movement generally. The chest exhibits the movement of front/back of the body, later swaying, and the descent of the trunk. Therefore, six wireless multi-function inertial sensors were placed on the patient, as shown in Figure 3. The joint angles of the patient at the elbows, shoulder, hip, and knees were measured using motion capture marker attached on the patient. 

## 3. Experiment

### 3.1. Purpose

The first purpose is to verify if utilizing only the measurements from the patient can exhibit the difference on a patient while the nurses conduct correct/incorrect skills. The second purpose is to employ the inertial sensor and confirm its applicability to the robot development in the future study. Accordingly, the following crucial points should be realized through this experiment.

- Confirm whether the translational acceleration, rotational speed, and joint angle of the patient can reveal the different influences on the patient’s movement while the nurse performs the correct and incorrect ways of assisting the patient.
- Determine the parameter and the location, which exhibits the most obvious difference on the patient between the correct and incorrect methods.
- Ensure the applicability of the inertial sensors to measure the translational acceleration and rotational speed.

### 3.2. Participants 

Two experienced nursing teachers were invited as participants in the experiments. The body mass and height of Teacher A was (180 cm/68 kg), and that of Teacher B was (169 cm/49 kg). Both nursing teachers have clinical experience in a hospital and experience in teaching the nursing skill of patient transfer. All procedures were approved by the Ethics Committee at the University of Tokyo, and all participants provided their written informed consent prior to enrollment.

### 3.3. Procedures

**Figure 4:**
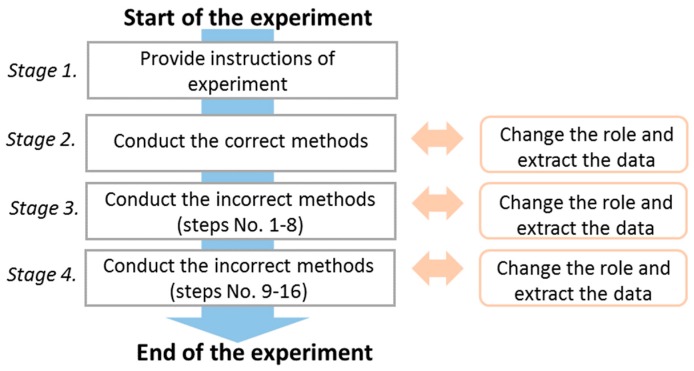
Experimental procedure.

There are four stages of the experiment, as shown in Figure 4. The first stage involves providing instructions regarding the experiment and installation of sensors and motion capture markers. During the experiment, one of the nursing teachers played the role of a patient affected by weak lower limbs, who required assistance during the transfer. A nursing teacher is ideal for such a role, as they have the clinical experience to accurately imitate such a patient. To shorten the experiment time required for attaching and removing the motion capture markers, both nursing teachers were attached with motion capture markers in the first stage whereas the teacher who simulated the role of the patient was also attached with inertial sensors. The second stage involved asking one nursing teacher to conduct the entire transfer skill from step No. 1 to 16 in the correct way in one trial as the nurse while the other nursing teacher simulated the role of the patient. Next, they switched their roles of nurse and patient and performed the transfer skill again. During the switching, the inertial sensors were placed on the nursing teacher who acted as the patient, and the data stored in the inertial sensors were simultaneously transferred to a personal computer via Bluetooth. In the third stage, both nursing teachers took turns to execute the incorrect skill listed from No. 1 to No. 9 in the checklist in one trial. Finally, both nursing teachers executed the incorrect ways listed in the checklist from No. 10 to No. 16 one after another. The checklist comprised 16 steps with correct/incorrect methods considering the limited experiment duration and the physical loading of nurses; hence, the experiment asked nursing teachers executing each correct/incorrect method for a single trial.

### 3.4. Experimental Setting

**Figure 5:**
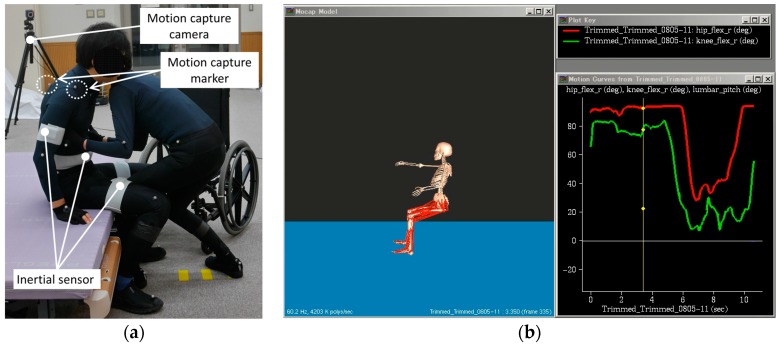
Illustration of the experiment (**a**) transfer trial (**b**) computation of joint angle of SIMM.

The environmental setting for the patient transfer included a bed and a wheelchair. The height of the bed was adjusted at 50 cm, which allowed the knees of the patient to bend to 90° when sitting on the bed. In addition, a wheelchair was placed at the end of the bed. Furthermore, to measure the patient’s movement, twelve motion capture cameras were placed around the bed. Two web cameras were utilized to record the front and side views, which were the reference to edit the motion capture trials. In addition, it is necessary to attach motion capture markers on the patient. A compression sportswear, pants, and gloves sewn with the motion markers were prepared and worn by the patient, which allowed the markers to remain firmly affixed on the patient. Furthermore, inertial sensors were attached on the patient’s arms, thighs, chest, and waist to measure the translational acceleration and rotational speed. The initial sensors were fixed on the body by wrapping them around the patient using elastic bandage with Velcro. The sensors and marker settings are described in previous session and shown in Figure 3. The *x*-axes of all inertial sensors were directed downward and their *y*-axes were directed toward the front side. And the illustration of experiment is shown in Figure 5.

### 3.5. Results

**Figure 6:**
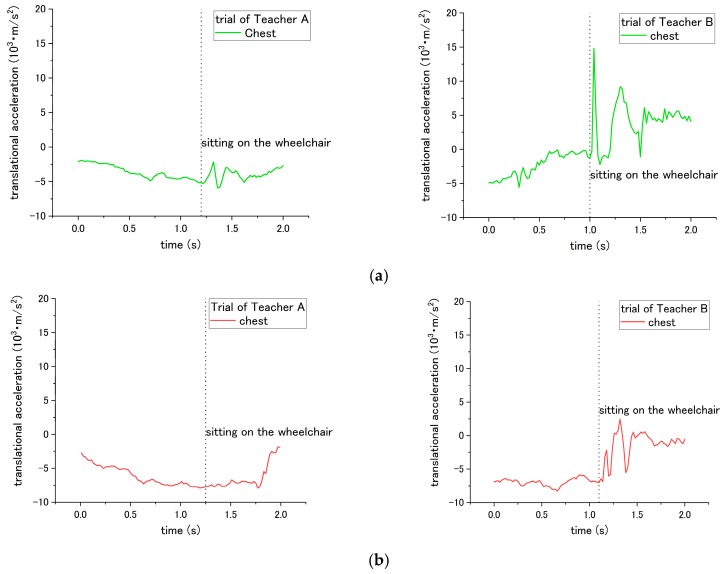
Translational acceleration of patient’s waist during step No. 15 under (**a**) correct way, and (**b**) incorrect way conducted at step No. 3.

All the data were obtained and analyzed by two-factor ANOVA, with two between factors of “*Method* (*correct*/*incorrect*)” and “*Teacher* (*A*/*B*)”. The experiment is illustrated in Figure 6. According to the result, the correct and incorrect ways given in the checklist influence the patient’s movement differently. This is consistent with the assumption made in our proposed method, i.e., three types of influences on the patient—translational acceleration, rotational speed, and joint angle—enable the evaluation of the skill of the nurse. Furthermore, the optimal location to measure the most obvious difference is presented in the data location. In addition, the descriptions of different influences of the measured data are listed in Table 1, which can be further used as the standard to evaluate the nursing skill. 

#### 3.5.1. Translational Acceleration

According to the results, the patient’s translational acceleration enables us to determine the difference between the correct and incorrect ways for steps No. 3, 14, and 15 of the checklist. For step No. 3, the correct way is applying the brake of the wheelchair; while the incorrect way is not applying the break. Thus, it caused a difference in translational acceleration during sitting down on the wheelchair in step No. 11. If the break was applied, during sitting down, the patient will be suddenly blocked by the chair-back and stop moving backward. In contrast, if the break was not applied, the wheelchair would start sliding backward after the patient sits down on it. Therefore, translational acceleration under the incorrect way did not fluctuate extremely compared with that under the correct way. As shown in Figure 6 and Table 2, the peak-to-valley of translational acceleration decreased from 2.35 × 10^3^ m/s^2^ to 1.10 × 10^3^ m/s^2^ when the incorrect way was conducted by Teacher A; the peak-to-valley of translational acceleration decreased from 9.54 × 10^3^ m/s^2^ to 8.53 × 10^3^ m/s^2^ under the incorrect ways of Teacher B.

**Figure 7:**
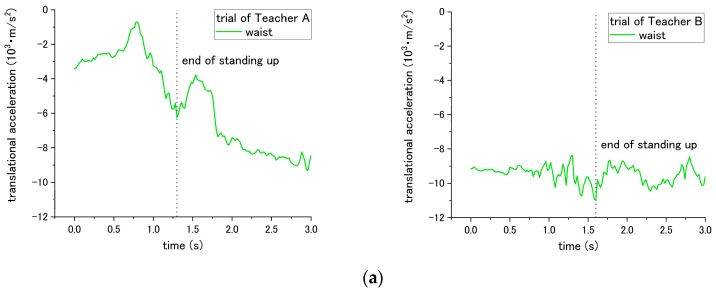

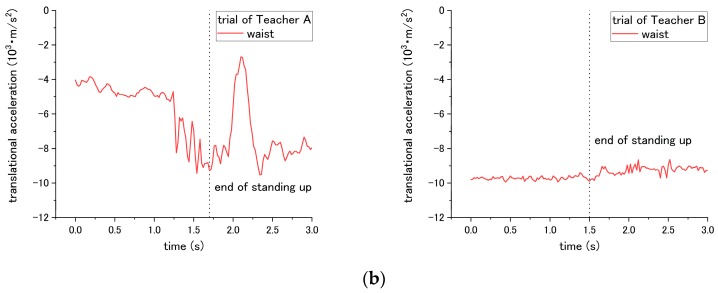
Translational displacement of patient’s waist during step No. 11 through (**a**) correct way, and (**b**) incorrect way conducted at step No. 10.

For step No. 10, the appropriate way for nurses is to squat down and lower their waist before assisting the patient to stand up. The incorrect way is standing up straight without lowering the waist. The difference in influence on the patient happens during the standing up at step No. 11. According to the results of Figure 7 and Table 2, when Teacher A did not lower his waist and then supported the patient to stand up, the peak-to-valley of translational acceleration increased from 5.53 × 10^3^ m/s^2^ to 6.58 × 10^3^ m/s^2^. However, for the trial of teacher B, the peak-to-valley of acceleration decreased from 2.59 × 10^3^ m/s^2^ to 1.57 × 10^3^ m/s^2^. Such inconsistent results on correct/incorrect ways were obtained and discussed in the following session. Hereafter, the peak-to-valley amplitude is considered as the change between the peak (highest value) and valley (lowest value, which can be negative), and value of peak/valley during the movement of standing up or sitting down.

**Figure 8:**
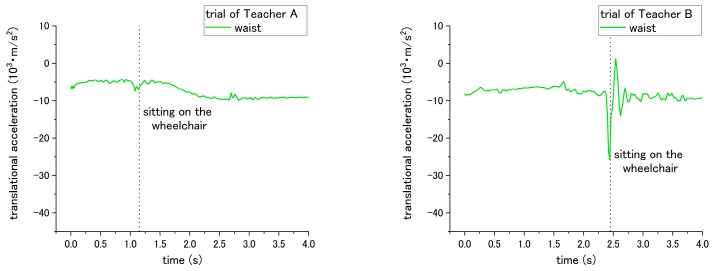
Translational acceleration of patient’s waist during step No. 15 through correct way.

**Figure 9:**
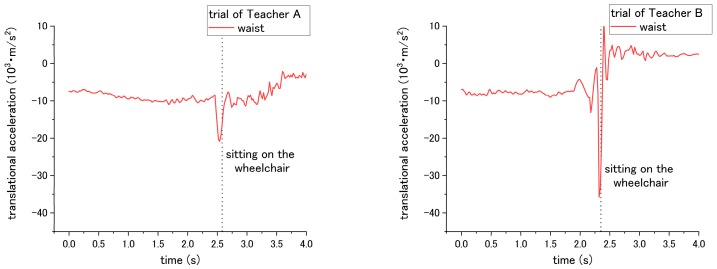
Translational acceleration of patient’s chest during step No. 15 through incorrect way conducted at step No. 14.

Both the correct and incorrect ways of step No. 14 and 15 are expected to have a different influence on the patient during the sitting down of step No. 15. Through the correct way of No. 14 and No. 15, the translational acceleration of patient during step No. 15 is shown in Figure 8. Step No. 14 is asking the nurses to lower their waist before making patient to sit down, while the incorrect way is to not lower their waist. The different influence is in terms of the translational acceleration of the patient’s waist during the sitting down. The correct way has smaller peak-to-valley of downward translational acceleration, while the peak-to-valley of downward translational acceleration becomes larger when the nurses do not lower their waist, as shown in Figure 9 and Table 2. 

**Figure 10:**
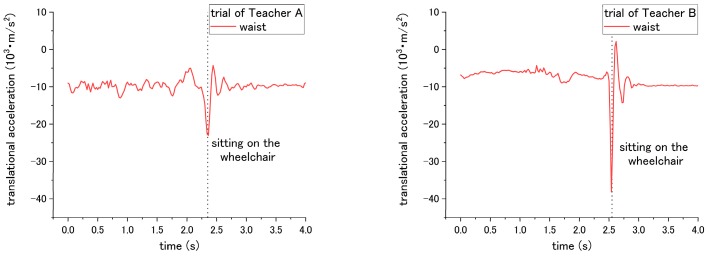
Translational acceleration of patient’s waist during step No. 15 when incorrect way was conducted at step No. 15.

For step No. 15, the appropriate way is making the patient lean forward first before assisting them to sit down, while the common mistake is making the patient to sit down straightly, making the patient’s trunk vertically move downward from standing condition to sitting. The translational acceleration of patient’s waist also exhibits huge difference between the correct and incorrect ways. Through the incorrect way, the peak-to-valley of translational acceleration becomes larger, increasing to 24.01 × 10^3^ m/s^2^ for the trial of Teacher A, and 40.28 × 10^3^ m/s^2^ for the trial of Teacher B, as shown in Figure 10 and Table 2. Although consistent results through the incorrect methods were obtained, according to the curves in Figure 7, Figure 8, Figure 9 and Figure 10, the peak-to-valley value also exhibited a difference on the patient between two nursing teachers. Accordingly, the factor has been elaborated in the Discussion section.

#### 3.5.2. Rotational Speed

According to the result, there are three steps No. 1, 5 and 9 in transfer skill that can be evaluated by the rotational speed. Step No. 1 is regarding the placed angle of the wheelchair. The correct way is placing the wheelchair around 20 to 30° with the bed, which requires the patient only to be rotated by a smaller angle to reach the position facing the wheelchair. On the contrary, the incorrect way is placing the wheelchair at an angle larger than 30°, forcing the patient to require a larger rotational angle to reach the wheelchair. Accordingly, the most obvious difference occurs in the rotational angle displacement, which is computed by multiplying the rotational speed and time, during the pivot turning step No. 12. The angle displacements of the waist are 100.4° and 96.1° for Teacher A and B, respectively, under the correct way, while the rotational angle displacement increased to 124.1° and 146.6° for Teacher A and B, respectively, under the incorrect way, revealing a significant difference (*p* = 0.046) as presented in Table 3. 

**Figure 11:**
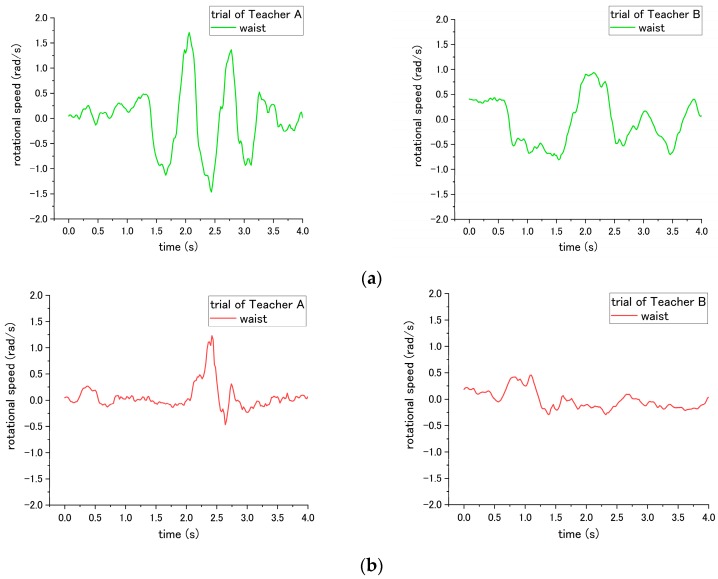
Rotational speed of patient’s waist during step No. 5 when the (**a**) correct way, and (**b**) incorrect way 5-(1) were conducted at step No. 5.

For step No. 5, the rotational speed reveals a difference in influence on the patient. This step is to move the patient on the edge of the bed by shifting the patient’s bottom. The incorrect way of No. 5-(1) is pulling the patient directly to the edge of bed without shifting the patient, which easily makes the patient fall down forward/backward. According to the results, the rotational speed of the waist varies from clockwise to counterclockwise alternately through the correct way, because the upper body of patient is rotated by the nurse. In contrast, the rotational speed did not show such behavior through the incorrect way, as shown in the Figure 11. In addition, the total rotational angle displacement exhibits a significant difference (*p* = 0.041) under correct and incorrect ways.

**Figure 12:**
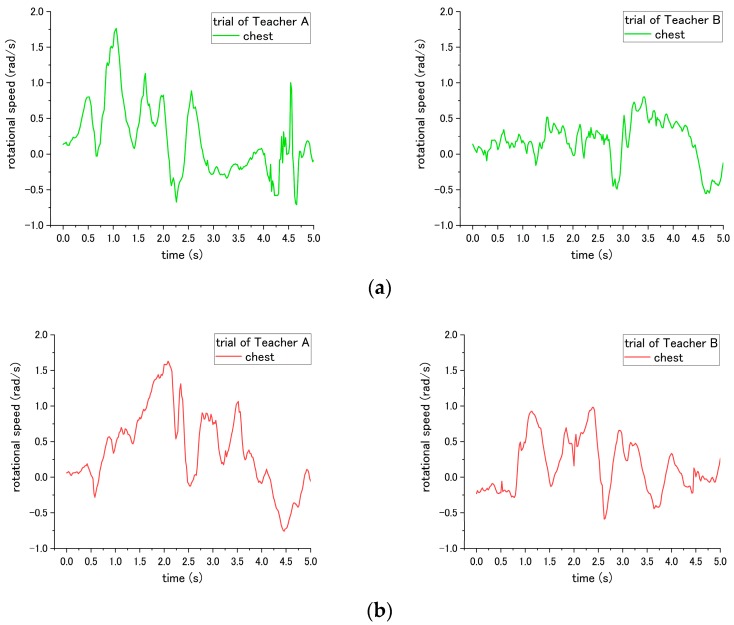
Rotational speed of patient’s chest during step No. 12 when the (**a**) correct way and (**b**) incorrect way were conducted at step No. 7.

Step No. 7, which requires the nurse to hug the patient before turning, was expected to cause a significant difference in influence on the patient during the pivot turning in step No. 12. However, a significant difference of the angular speed of patient was difficult to be found in both teachers. The results of the rotational speed of patient’s chest during step No. 12 are shown in Figure 12. For the trials of Teacher A, the MAX-to-MIN of rotational speed of patient is 2.47 rad/s under the correct way, and decreases to 2.38 rad/s under incorrect ways. On the other hand, for Teacher B, the MAX-to-MIN of rotational speed is 1.36 rad/s under the correct way, and increases to 1.57 rad/s under the incorrect way. Potential reasons contributing to such inconsistent results are presented in the next section of the discussion. 

**Figure 13:**
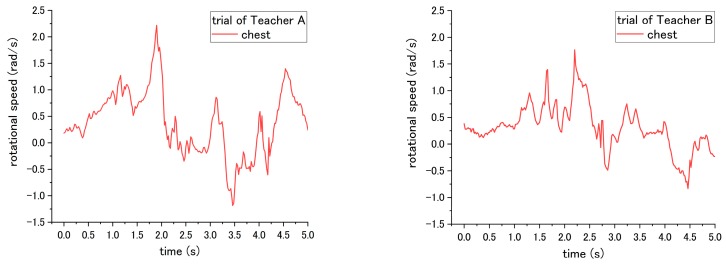
Rotational speed of patient’s chest during step No. 12 when the incorrect way was conducted at step No. 9.

For step 9, the correct way is asking the nurses to place their right leg behind them, and put their left leg between the patient’s two feet, while the incorrect way is placing their feet in an opposite position. The difference in influence on patient occurs in step No. 12 during the pivot turning, as shown in Figure 13. The MAX-to-MIN rotational speed increased from 2.47 rad/s to 3.40 rad/s for teacher A under the incorrect way, and for teacher B, the rotational speed increased from 1.36 rad/s to 2.60 rad/s when employing the incorrect way.

#### 3.5.3. Joint Angle

**Figure 14:**
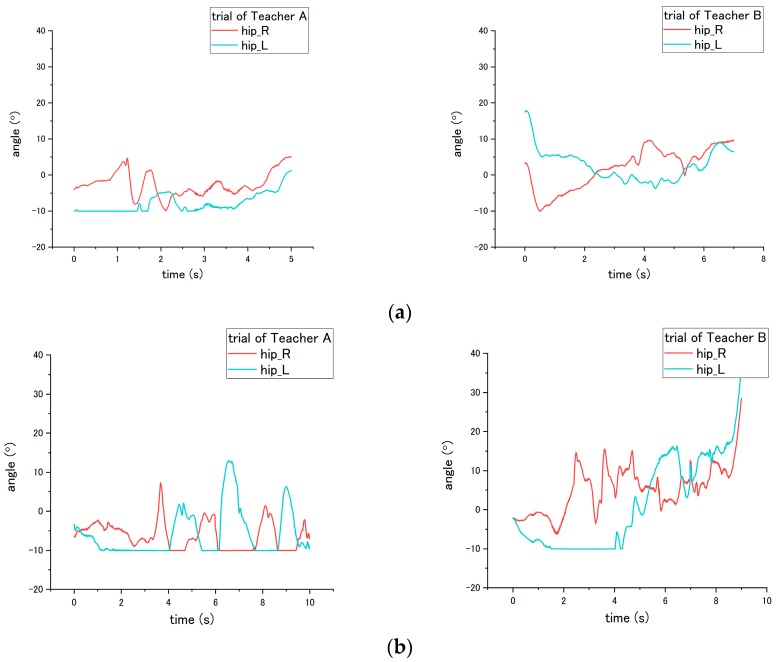
Flexion angle of patient’s hip during step No. 11 through the (**a**) correct way, and (**b**) incorrect way conducted at step No. 2.

With regard to the joint angle, such movement on a patient enables us to evaluate the correct and incorrect ways of seven steps (No. 2, 5, 6, 7, 11, 15 and 16) in the transfer skill. Step No. 2 is related to the distance from the bed to the wheelchair, settled by the nurse. However, the translational displacement is not measured in this study; therefore, to evaluate the correct and incorrect ways in this step, we probed the joint angle instead. For step No. 2, the appropriate way is to place the wheelchair close to the bed, while the inappropriate is to place the wheelchair too far away from the bed. According to the results, the obvious difference in influence on the patient occurred on the hip joint angle because the patient needs to move farther with more walking paces to reach the wheelchair under the incorrect ways. As shown in Figure 14, a variation between the flexion and extension angle represents the pace of the patient while walking. Moreover, as presented in Table 4, the average angle displacement of Teacher A and B is 81.7° under the correct way, while 189.3° under the incorrect way, exhibiting a significant difference (*p* = 0.047) between correct and incorrect ways.

**Figure 15:**
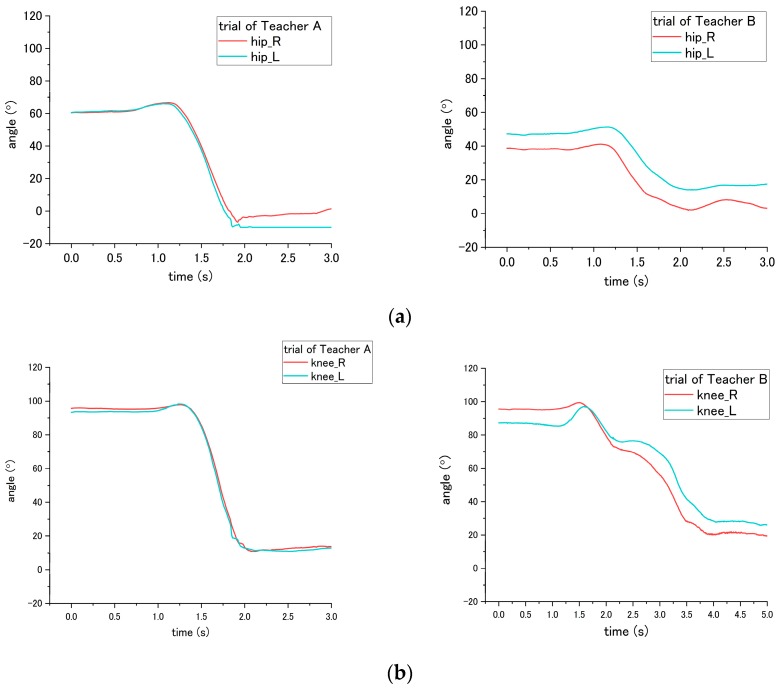
Flexion angle of patient’s (**a**) hip and (**b**) knee during step No. 11 when correct way was conducted at steps No. 5, 6 and 11.

**Figure 16:**
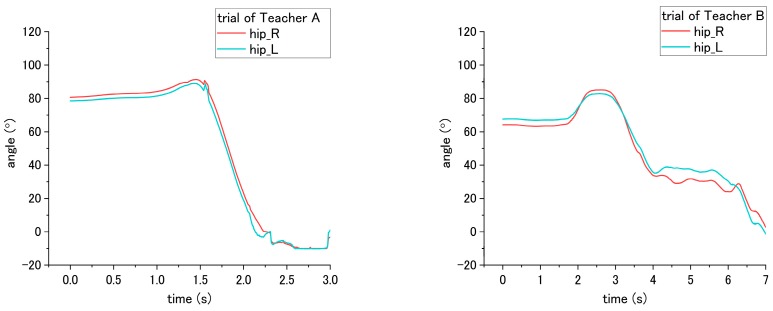
Flexion angle of patient’s hip during step No. 11 when the incorrect way was conducted at step No. 5-(2).

The incorrect ways of step No. 5, 6, and 11 affected the joint angle of patient during standing up at step No. 11. To perform analysis through comparison, the variation angles of the knee and hip when the correct way was conducted by nurses, are presented in Figure 15. For step No. 5, the correct way is to move the patient to the edge of the bed, while the incorrect way of 5-(2) is not executing this step, making the patient not move to the edge of bed. The difference in influence on the patient between correct and incorrect ways occurs on the hip joint angle during the standing up at step No. 11. Through the incorrect way, the average peak angle increased from the 66.3° to 90.2° for trial of Teacher A, while the average peak angle increased from 46.2° to 84.0° for the trial of Teacher B, as shown in Figure 15a and Figure 16. The peak angle (*p* < 0.001) and the variation from initial-to-peak (*p* = 0.003) exhibit significant differences under correct and incorrect ways. Furthermore, the peak angle of the knee also shows a significant difference (*p* = 0.008) between Teacher A and B, as presented in Table 4.

**Figure 17:**
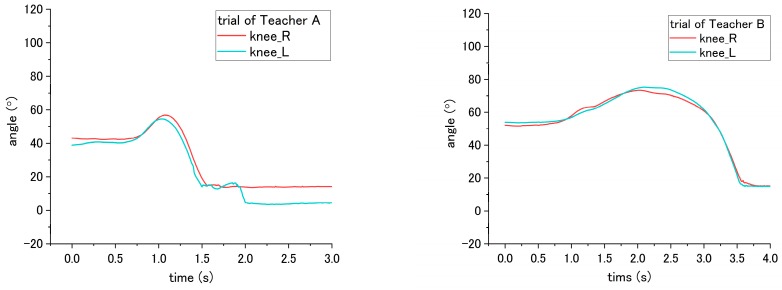
Flexion angle of patient’s knee during step No. 11 when incorrect way was conducted at step No. 6.

Step No. 6 is to move the patient’s ankle close to the bed, and the incorrect way is moving the patient’s ankle too far away from the bed. Those ways lead to the difference in step No. 6 and also the standing up step No. 11. According to Figure 15b, the initial angle of the knee in the figure represents the angle of a patient after their ankles were moved to bedside. When placing the ankle too far away from the bed, the knee angle of the patient became smaller; accordingly, the incorrect and correct ways reveal a significant difference (*p* < 0.001) on the knee angle. In addition, during the standing up at step No. 11, the peak angle (*p* < 0.001) and the variation angle of initial-to-peak (*p* = 0.02) also reveal a significant difference between correct/incorrect ways, as shown in Figure 17. Furthermore, a significant difference was found between Teacher A/B at the peak angle (*p* < 0.001) and variation angle of initial-to-peak (*p* = 0.045) of the knee joint.

**Figure 18:**
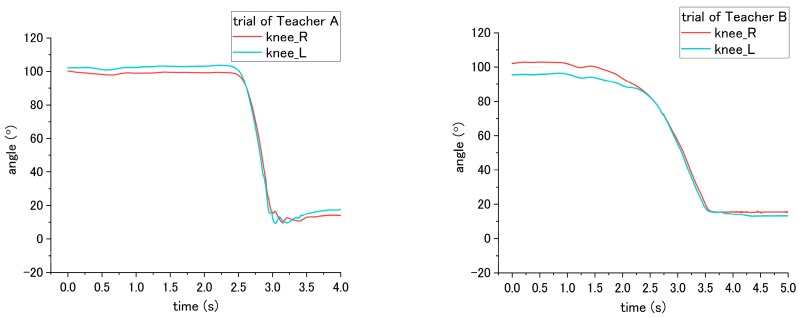
Flexion angle of patient’s knee during step No. 11 when the incorrect way was conducted during step No. 11.

**Figure 19:**
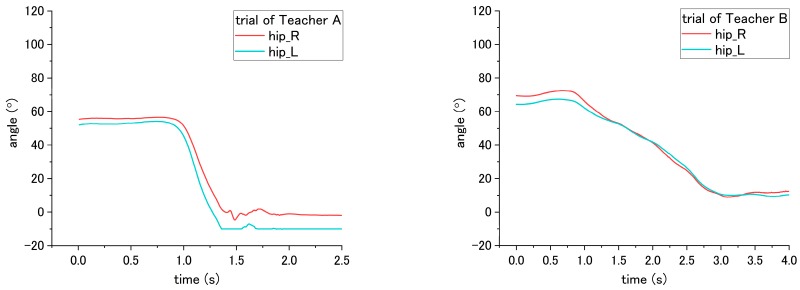
Flexion angle of patient’s hip joint during step No. 11 when incorrect way was conducted at step No. 11.

For step No. 11, i.e., assisting the patient to stand up, the correct way is to make the patient lean forward first before supporting them to stand up, while the incorrect way is to hold the patient to stand up vertically. According to the result shown in Figure 15b, the knee angle increases first, and then decreases during standing up through the correct way, while through the incorrect way, i.e., that the nurse supporting the patient upward vertically without making them lean forward, the knee angles do not increase obviously in the beginning, as shown in Figure 18 and Figure 19. The variation of initial-to-peak angle became smaller while the incorrect ways were conducted, and a significant difference (*p* = 0.049) was found. At the same step of No. 11 during standing up, the nurse’s correct/incorrect ways also led to a significant difference (*p* = 0.005) in the initial-to-peak angle of hip joint. However, in the trials of Teacher B, the patient’s hip angle increased at the beginning, which differs from decreasing directly in the case of Teacher A, as shown in Figure 19. The potential reason is presented in the discussion section.

**Figure 20:**
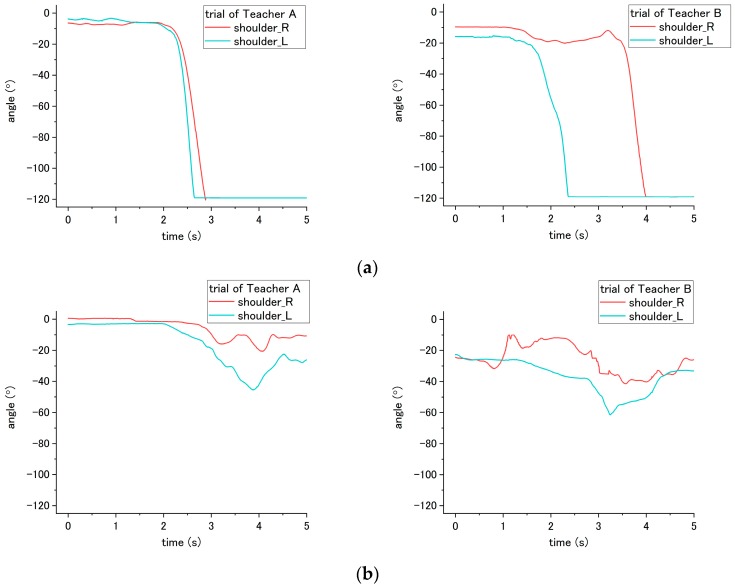
Adduction angle of patient’s shoulder during step No. 7 through the (**a**) correct way, and (**b**) incorrect way conducted at step No. 7.

During step No. 7, the nurses are asked to place the patient’s arm around their shoulder and hug them; therefore, when the correct way was applied, the patient arm will raise and fold to hug the nurses. Thus, according to the result of Figure 20, the adduction shoulder angle decreased when hugging the nurse, and the variation angle from initial-to-valley is larger than the incorrect way when the patient’s arms would not be moved to hug the nurses. The result shows a significant difference (*p* < 0.001) between the correct and incorrect methods.

**Figure 21:**
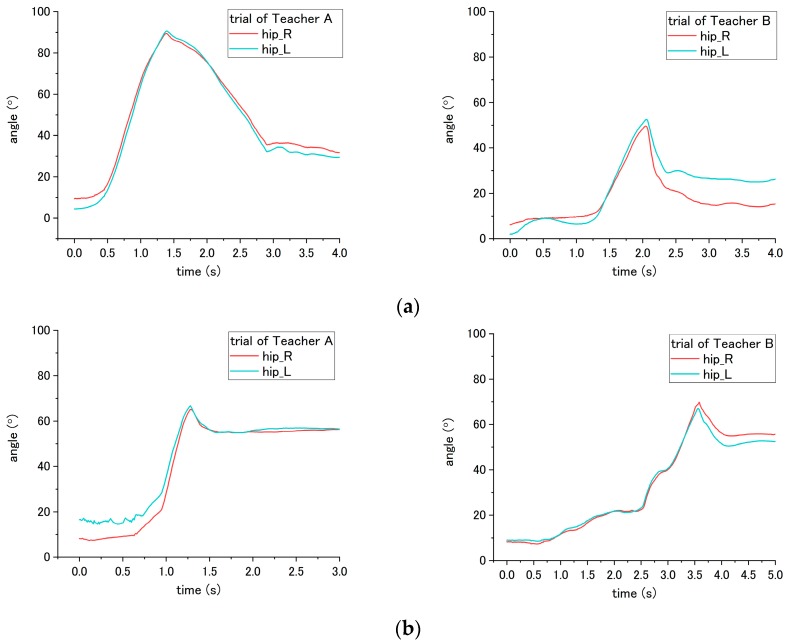
Flexion angle of patient’s hip during step No. 15 when the (**a**) correct way, and (**b**) incorrect way were conducted at step No. 15.

In terms of step No. 15, the appropriate way is making the patient lean forward first before assisting them to sit down, while the common mistake is making the patient sit down on the wheelchair by moving the patient’s trunk vertically downward from standing condition to sitting. In Figure 21, the result for the correct way shows the hip angle increasing until the patient sits on the seating surface of wheelchair, and then the angle started to decrease until the end. However, the results for the incorrect way did not show such an obvious decreasing trend after the patient sits on the wheelchair. The peak-to-end angle under the correct ways is 59.5° for Teacher A and 30.25° for Teacher B, while the peak-to-end angle is 9.55° for Teacher A and 14.45° for Teacher B under the incorrect way, which exhibits a significant difference (*p* < 0.001) between correct/incorrect ways. In addition, a significant difference (*p* = 0.005) was also found between Teacher A and B.

**Figure 22:**
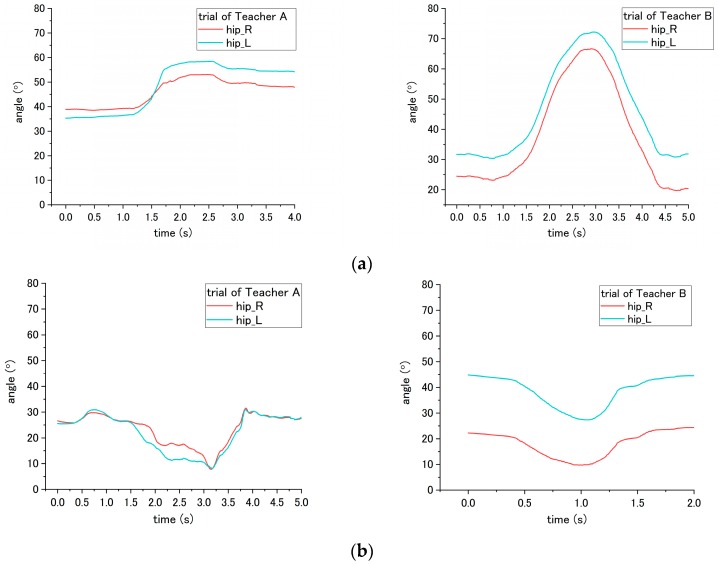
Flexion angle of patient’s hip during step No. 16 when the (**a**) correct way, and (**b**) incorrect way were conducted at step No. 16.

The last step, No. 16, is adjusting the patient’s posture and making the patient sit against the backrest of wheelchair. The correct way is to hold the patient’s both arms and make them lean forward before pulling them to sit against the backrest. In contrast, the incorrect way is holding the patient’s armpits and lifting them upward, and finally moving them to lie on the backrest. The difference in correct and incorrect ways was shown on the hip angle. The hip angle first increased to a peak value when the correct was applied, as shown in Figure 22a, while through the incorrect way, the hip angle decreased to the valley value, as shown in Figure 22b. A significant difference (*p* = 0.005) in the peak/valley value was found. 

## 4. Discussion

To evaluate the nurse’s skill by measuring only the moment of patient, the translational, rotational, and joint angles were observed and determined in the experiment. Based on the results, a three-step checklist was determined to be able to reveal the difference between the correct and incorrect methods through translational acceleration. Additionally, by using rotational speed, the difference can be determined using three steps. Finally, the joint angle reveals the difference between the correct and incorrect methods conducted by nurses in seven steps. A more detailed description is presented below.

### 4.1. Translational Acceleration 

The translational acceleration of a patient the evaluation of the correct and incorrect methods for three steps during the transfer of skill. Steps 3, 14, and 15, influence the patient’s movements while standing up or sitting down. These results show that the dynamic movement of the patient becomes more rapid when the support by the nurse is inappropriate.

As shown in Table 2, incorrectly performing the different steps may cause the same effect on the patient’s movement. For example, incorrectly performing steps No. 14 and 15 causes the absolute value of acceleration to become larger. Accordingly, to distinguish which step was performed incorrectly, the flexion angle of the hip joint needs to be considered. If step No. 15, in which the nurse assists the patient in sitting down without making them leaning down is performed incorrectly, the hip joint angle increases at first but does not show a clear decrease after sitting on the wheelchair as shown in Figure 21b. While if step No. 14 is performed incorrectly but No. 15 is performed correctly, then the flexion of hip joint angle will increase first and then decreases while sitting down, as shown in Figure 21a.

According to the results from step No. 10, the translational acceleration did not show consistent variation between the correct incorrect executions. The correct method is to ask the nurses to lower their waist before helping the patient to stand up, while the incorrect method involves assisting the patient in standing up without lowering their waist first. For Teacher A, the peak-to-valley of acceleration increased when the step was performed incorrectly. However, for Teacher B, the acceleration decreased when the step was executed incorrectly. In addition, a significant difference was found between Teacher A and B, revealing that the translation acceleration of the patient has a strong effect on the teachers, as shown in Figure 7. A reason for this may be because Teacher B’s simulated patient, who is simulated by Teacher A (weight 68 kg), is heavier than Teacher B (weight 49 kg). This makes it difficult for Teacher B to move the patient’s body instantly, and Teacher B must exert a greater force to assist the patient in standing up. Accordingly, even though Teacher B did not lower the waist before assisting the patient in standing up, it is difficult for Teacher B to rapidly lift the patient because of the significant weight difference. Similar with the results under the incorrect methods in step nos. 14 and 15, both Teachers A and B revealed a consistent influence on the patient, which caused a larger translational acceleration. However, the acceleration of the patient was larger in the trial of Teacher B. Such a result was also associated with the different simulated patient’s weight. Teacher B’s simulated patient, who was reproduced by Teacher A, was heavier than Teacher A’s simulated patient. The heavier weight of the simulated patient brought a larger downward acceleration while the nurses assisted the patient in sitting down on the wheelchair through the incorrect methods. Such an issue of individual difference on the simulated patient can be solved by regulating the simulated patient with a normalized weight. When a robot patient is developed, this issue will also be erased because only a single simulated patient is reproduced by the robot.

### 4.2. Rotational Speed 

Rotational speed enables the evaluation of steps. 1, 5, and 9. The incorrect execution of the aforementioned steps contributes to the different rotational speed on the patient. There are six inertial sensors attached to different parts of the patient’s body. These sensors are used to measure the optimal data to evaluate the nurse’s skills; the details are discussed below. 

For step No. 1, related to the angle of the wheelchair, the influence on the patient is the rotational angle of the patient while being moved from the bed to the wheelchair. The rotational speed of the waist was determined to compute the rotational angle. This is because, compared with other body parts, such as the chest or arm, the waist is not easily affected by the movement of arm and the upper body. Accordingly, the data measured at the waist accurately reveal the rotational angle of the patient. Similarly, with step No. 5, the difference between the correct and incorrect execution is a shifting movement of the patient’s bottom. Therefore, the rotational speed of the waist, which is closest to the bottom, can be used to determine the rotation of the bottom. For steps 7 and 9, the purpose is to determine if the rotational speed of the upper body is more strenuous while turning. Therefore, the location of measurement is the chest instead of the waist. Thus, motions such as instable swaying during pivot turning can be observed.

In Table 3 of step No. 5, both nurses’ trials exhibited consistent results of the total angle displacement being larger under the correct method than the incorrect method. Figure 11 shows that the curves of both Teachers A and B revealed a difference in the rotational speed of the patient. The results were caused by the variation of the correct/incorrect methods existing among different individuals. For example, the correct method is to enable the patient to sit on the edge of the bed by shifting the patient’s bottom. Teachers A and B used different levels of shifting the patient’s bottom. Teacher A rotated the patient’s bottom with a larger angle displacement than Teacher B. In the incorrect method, the patient was moved to the edge of the bed without shifting the patient’s bottom. Teacher A supported the patient’s bottom and pulled the patient directly to the edge of the bed with the impulse that changed the patient’s direction a little at the beginning. This led to the sudden change in the rotational speed. Teacher B smoothly held and moved the patient to the edge of the bed smoothly without shifting. Such variation contributed to the different influence on the patient. This result suggested that the threshold should be decided as the angle variation computed by the rotational speed instead of the curve or peak value of the raw data. For step No. 7, the incorrect execution, in which is the nurse did not perform a mutual hug with the patient during the pivot turning, did not reveals consistent differences between Teacher A and B. When executed incorrectly, the patient turned by Teacher A had a lower rotational speed, whereas the patient turned Teacher B had a higher rotational speed. The results may be attributed to the following factors. First, Teacher B is shorter and lighter than Teacher A; therefore, when Teacher A did not perform a mutual hug with the patient simulated by Teacher B, the upper body become more unstable and had a more acute rotational speed. However, when Teacher B turned the patient that was simulated by Teacher A, the unstable rotational speed was not prominent.

Based on the results, using the translational acceleration enables the evaluation of step No. 5 (related to shifting of the patient’s bottom) and also step No. 12 (related to pivot turning). Among these steps, the translation motion is crucial to determining the correct and incorrect execution. 

### 4.3. Joint Angle 

According to the results, using the joint angle enables the evaluation of seven steps (2, 5, 6, 7, 11, 15, and 16) among the transfer skill. These results also reveal that the joint angle is the most vital parameter in evaluating the nursing skill during the transfer. The angles involved include the abduction-adduction of the shoulder and flexion-extension of both the hip and the knee. The angles of the hip and the knee have different influences on the patient while being incorrectly assisted by nurses in standing up and sitting down. Furthermore, the angle of the shoulder reveals the differences related to mutual hugging for step No. 7. Additionally, as shown in Table 4, significant differences in patient’s joint angle were found between correct and incorrect execution. 

However, the significant differences were also found between Teacher A and B (steps 5, 6, 7, and 15). The reason for this result may because of the different heights of patients that affect the joint angle. There are two participants, Teacher A and B. Teacher A is 180 cm, and is taller than Teacher B (164 cm). During the experiment, they take turns in functioning as the nurse and patient. When Teacher A performed the procedures, Teacher B simulated the patient. An example of this can be found in the sitting posture, as shown in Figure 15. When sitting on the bed, the patient simulated by Teacher A (180 cm) has a larger flexion angle of the knee than the patient stimulated by Teacher B (164 cm). This length leads to a larger blended angle of flexion when sitting on the bed. Despite this issue (caused by individual differences), the joint angles still reveal consistent variation and significant differences between correct and incorrect execution of the steps. 

Similarly, other issues related to both the patient’s height and the nurse’s height is raising in step No. 11. Under the trials of Teacher A, the correct and incorrect execution caused the same angle variation on the patient’s hip angle, which first increased and then decreased. For step No. 11, the correct method is to lean forward the patient’s trunk before the standing up. The incorrect method is to vertically to lift up the patient’s trunk. For the trial of Teacher B, because the patient who simulated by Teacher A is taller than Teacher B, vertically lifting the patient became difficult. Accordingly, after mutual hugging with the patient, it is inevitable for Teacher B to incline the patient’ trunk forward and then support the patient in standing up. The incorrect method is shown in Figure 19. The hip joint angle first increased due to the forward incline of the patient’s trunk. The issues regarding height differences between the patient and nurse can be solved by the following solution. First, develop the robot patient with a fixed height; next, recruit the patricians with normalized height.

Apart from those three parameters, the applicability of the inertial sensor was verified according to the results of the translational acceleration and the rotational speed. Therefore, the inertial sensor will be developed on the robot’s waist and chest parts. In the future work, the joint angle measured by motion capture will be replaced by the encoder developed on the robot. Compared with other methods requiring an algorithm or a computation (i.e., inertial motion capture uses IMUs), using the encoder is the most direct method of measuring the angle with a relatively high precision. It is also the reason why this study only emphasized the verification of the applicability on the inertial sensor.

## 5. Conclusions and Future Work

With the goal of evaluating the transfer skill of nurses by measuring the patient, this study observed the translational acceleration, rotational speed, and joint angle to investigate if the difference in the patient under correct/incorrect methods can be obtained. The applicability of inertial sensors was verified as a pre-work of robot development because in the future, inertial sensors will be installed on the robot to measure the translational acceleration and the rotational speed. During the experiment, the motion capture was employed to measure the joint angle, which will be replaced by the encoder on the robot. A checklist consisting of 16 steps with the correct and incorrect execution methods and common mistakes were proposed by nursing teachers. Additionally, the steps that may cause different influences on patients, depending on whether the step was correctly or incorrectly executed, were listed in the checklist. An experiment was conducted in which nursing teachers were asked to transfer a patient using both the correct and incorrect method. The results obtained from the experiment are summarized as follows:

- The translation acceleration can be used to reveal the differences in the patient’s kinematic movement (i.e., fast or slow) between the correct and incorrect executions in step nos. 3, 14, and 15. The difference in step No. 10 is difficult to exhibit.
- The rotational speed enables showing the influence on the patient in step nos. 1, 5, and 9, which are related to shifting the patient’s bottom and pivot turning. In step No. 7, the expected difference between the correct/incorrected methods is not obtained.
- The joint angle shows a significant difference in the patient in most steps, partially for the steps of standing up and sitting down. A significant difference is also observed between different nurses.
- The differences between the patient’s and the nurse’s height would influence the joint angle. The differences in the patient’s weight would affect the translation acceleration. However, consistent results are still obtained from different nurses under the incorrect methods.
- The threshold of each parameter should be determined considering the individual differences of the nurse’s and the simulated patient’s weight and height.
- The applicability of inertial sensors was verified for use in the robot’s development.

According to the results, the three parameters are able to exhibit the difference under the correct/incorrect methods; thus, the next step is to determine the threshold. The threshold to evaluate the correct/incorrect execution of each step will be determined by inviting nurses with a normalized height and simulated patients with a normalized weight to make the proposed method usable in the general situation of nursing education. Furthermore, some post-processes (i.e., FIR filter) will be employed to reduce the effect of noise on raw data, enabling the determination of more precise thresholds. Lastly, other experiments with the nursing trainees will be conducted to verify if the proposed system and the determined threshold are applicable. Also, in the future, a robot patient that can be used to measure the translational acceleration, rotational speed, and joint angle, will be developed. Thus, the evaluation system without the supervision of a teacher and any sensor setting on learners and environments can be achieved. 

## Figures and Tables

**Table 1:** Check list of patient transfer skill.

Step of Transfer Skill	Correct Method	Incorrect Method	Influence on Patient Occurs in Step(s)	Parameter Type	Data Location	Different Influences on the Patient under Correct/Incorrect Methods
No. 1	Place the wheelchair at the bedside and adjust the angle to 20–30°	Place the wheelchair at the bedside at a very large angle	No. 12	Rotational speed	Waist	The rotational angle computed by the rotational speed becomes larger when the incorrect method is applied during No. 12
No. 2	Place the wheelchair near the bed	Place the wheelchair very far from the bed	No. 12	Joint angle	Hip	The total variation of the joint angle increases while moving the patient from the bed to the wheelchair when the incorrect method is applied in No. 12
No. 3	Apply the wheelchair brakes	Do not apply the brakes	No. 15	Translational acceleration	Chest	Under the correct method, backward acceleration forms a peak with sudden increasing/decreasing caused by stopping of the wheelchair in No. 15
No. 4	Place one of your feet behind you and another foot between the feet of the patient	—	—	—	—	—
No. 5	Enable the patient to sit on the edge of the bed by shifting the patient’s bottom	5-(1) Move the patient to the edge, but not by shifting the patient’s bottom	No. 5	Rotational speed	Waist	Repeated variation in the rotational speed between the clockwise and counterclockwise directions when the correct method is applied in No. 5
		5-(2) Do not move the patient to the edge of the bed	No. 11	Joint angle	Hip	The joint angle obviously increases while starting to stand up when this step is not executed in No. 11
No. 6	Adjust the patient’s leg posture and move the patient’s ankle closer to the bed	Move the patient’s ankle far from the bed	No. 6	Joint angle	Knee	The joint angle decreases when the ankle is placed very far from the bed in No. 6
			No. 11		Hip	The joint angle increases at the beginning of standing up when the ankle is placed very far from the bed in No. 11
No. 7	Place both arms of the patient on your shoulders and hug	Do not place both arms of the patient on your shoulders	No. 7	Joint angle	Shoulder	The adduction angle decreases when the nurse raises the patient’s arm and hugs the patient in No. 7 under the correct method
			No. 12	Rotational speed	Chest	Non-consistent results of teachers A and B are found during the pivot turning in No. 12
No. 8	Clutch the lower back of the patient.	—	—	—	—	—
No. 9	Place your right foot behind you and the left foot between the feet of the patient	Place your feet in the wrong position: left foot behind and right foot between the feet of the patient	No. 12	Rotational speed	Chest	The rotational speed increases when the incorrect method is applied in No. 12
No. 10	Squat down and lower your waist to prepare the patient to stand up	Do not bend your knees and lower your waist	No. 11	Translational acceleration	Waist	The upward translational acceleration increases during standing up when the incorrect method is applied in No. 11
No. 11	Make the patient lean forward, then assist the patient to stand up	Do not make the patient lean forward first; make them stand up vertically	No. 11	Joint angle	Hip	The joint angle increases at the beginning of the standing movement when the correct method is applied in No. 11, but Teacher A’s trial did not obtain such a result on the hip angle
					Knee	
No. 12	Use your left foot as a pivot axis to help the patient turn to the wheelchair	—	—	—	—	—
No. 13	Place one of your feet behind you and another foot between the feet of the patient	—	—	—	—	—
No. 14	Lower your waist to prepare assisting the patient to sit down	Do not lower the waist and bend the knee to assist the patient to sit down	No. 15	Translational acceleration	Waist	The downward translational acceleration increases when the nurses do not lower their waist in No. 15
No. 15	Make the patient lean forward and assist the patient to sit	Do not make the patient lean forward first before making them sit down	No. 15	Joint angle	Hip	The joint angle of the hip increases, then decreases when the correct method is applied in No. 15
				Translational acceleration	Waist	The downward translational acceleration increases when the incorrect method is applied in No. 15
No. 16	Make the patient sit in the wheelchair by pulling with both arms	Lift the patient up vertically and make the patient sit in the wheelchair	No. 16	Joint angle	Hip	The joint angle decreases at first when the incorrect method is applied and increases when the correct method is applied in No. 16

**Table 2:** Translational acceleration in translational acceleration of patient.

Step	Influence on Step	Value (Unit)	Correct Way		Incorrect Way	
			Teacher A	Teacher B	Teacher A	Teacher B
No. 3	No. 15	peak-to-valley (10^3^ m/s^2^)	2.35	9.54	1.10	8.53
No. 10	No. 11	^┬^ peak-to-valley (10^3^ m/s^2^)	3.33	1.93	4.62	0.69
No. 14	No. 15	peak-to-valley (10^3^ m/s^2^)	3.13	26.93	18.65	47.78
No. 15	No. 15	peak-to-valley (10^3^ m/s^2^)	3.13	26.93	24.01	40.28

^┬^ Non-consistent influence between correct and incorrect way.

**Table 3:** Results of rotational speed.

Step	Influence on Step	Value (Unit)	Correct Way		Incorrect Way	
			*Teacher A*	*Teacher B*	*Teacher A*	*Teacher B*
No. 1	No. 12	* Total angle variation (°)	100.4	96.1	144.1	146.6
No. 5	No. 5	* Total angle variation (°)	105.2	108.6	*No. 5-(1)* 56.1	*No. 5-(1)* 52.8
No. 7	No. 12	^┬^ MAX-to-MIN (rad/s)	2.47	1.36	2.38	1.57
No. 9	No. 12	MAX-to-MIN (rad/s)	2.47	1.36	3.40	2.60

^┬^ Non-correlated influence between correct and incorrect ways. * Significant difference between correct and incorrect ways (*p* < 0.05).

**Table 4:** Results of joint angle.

Step	Influence on Step	Value (Unit)	Correct Way				Incorrect Way			
			*Teacher A*		*Teacher B*		*Teacher A*		*Teacher B*	
			*R*	*L*	*R*	*L*	*R*	*L*	*R*	*L*
No. 2	No. 12	* Total angle displacement (°)	102.3	55.2	95.2	73.9	146.7	156.3	298.2	156.1
No. 5	No. 11	*^▲^ Hip peak angle (°)	66.6	66.0	41.1	51.3	*No. 5-(2)*			
							91.4	89.0	85.1	82.9
		* Hip int. to peak angle (°)	6.2	5.5	2.5	4.1	*No. 5-(2)*			
							10.8	10.5	21.0	15.3
No. 6	No. 6	* Knee int. Angle (°)	95.6	93.3	95.5	87.2	43.1	38.9	52.1	53.9
	No. 11	*^▲^ Knee peak angle (°)	97.8	98.3	99.4	97.2	56.9	54.4	73.3	75.2
		*^▲^ Knee int. to peak angle (°)	2.6	4.9	3.8	9.8	13.8	15.6	21.2	21.3
No. 11	No. 11	* Hip int. to peak angle (°)	6.2	5.5	3.5	4.1	0.8	1.1	3.2	2.7
		* Knee int. to peak angle (°)	2.6	4.9	3.8	9.8	0	1.2	0.7	0.9
No. 7	No. 7	* Shoulder int. to valley (°)	114.0	115.3	109.6	103.3	21.2	42.2	16.99	38.7
No. 15	No. 15	*^▲^ peak to end (°)	57.8	61.2	34.2	26.3	8.9	10.2	14.2	14.7
No. 16	No. 16	* Peak/valley angle (°)	53.1	58.4	66.7	72.2	7.8	8.1	9.7	27.3

* Significant difference between correct and incorrect ways (*p* < 0.05); ^▲^ Significant difference between Teacher A and B (*p* < 0.05).
